# Prevention of radiotherapy-related oral mucositis with zinc and polyherbal mouthwash: a double-blind, randomized clinical trial

**DOI:** 10.1186/s40001-023-01015-8

**Published:** 2023-03-02

**Authors:** Marzieh Sahebnasagh, Vahideh Aksi, Fatemeh Eslami, Hossein Lashkardoost, Jamal Kasaian, Shiva Golmohammadzadeh, Bahareh Parkam, Reza Negarandeh, Fatemeh Saghafi, Adeleh Sahebnasagh

**Affiliations:** 1 School of Dentistry, North Khorasan University of Medical Sciences, Bojnurd, Iran; 2 Student Research Committee, School of Dentistry, North Khorasan University of Medical Sciences, Bojnurd, Iran; 3 School of Public Health, Addiction & Behavioral Research Center, North Khorasan University of Medical Sciences, Bojnurd, Iran; 4 Natural Products and Medicinal Plants Research Center, North Khorasan University of Medical Sciences, Bojnurd, Iran; 5 Nanotechnology Research Center, Pharmaceutical Technology Institute, Mashhad University of Medical Sciences, Mashhad, Iran; 6 Department of Pharmaceutics, Faculty of Pharmacy, Mashhad University of Medical Sciences, Mashhad, Iran; 7 Imam Ali Hospital, North Khorasan University of Medical Sciences, Bojnurd, Sari Iran; 8 Student Research Committee, Department of Pharmaceutics, Faculty of Pharmacy, Mazandaran University of Medical Sciences, Sari, Iran; 9 Department of Clinical Pharmacy, School of Pharmacy, Shahid Sadoughi University of Medical Sciences and Health Services, Yazd, Iran; 10 Clinical Research Center, Department of Internal Medicine, School of Medicine, North Khorasan University of Medical Sciences, Bojnurd, Iran; 11 Department of Surgical Medicine, Faculty of Medicine, North Khorasan University of Medical Sciences, Bojnurd, Iran

**Keywords:** Radiotherapy, Oral mucositis (stomatitis), Mouthwash, Head and neck cancer, Zinc sulfate, Polyherbal, Prevention

## Abstract

**Background:**

A significant percentage of head and neck cancer (HNCs) patients receiving RT experience oral mucositis (OM). This study aimed to evaluate the effect of the polyherbal (containing chamomile, peppermint oil, *Aloe vera*, and honey) and zinc mouthwashes in comparison to the control (chlorhexidine) and placebo groups for prevention of radiation-induced OM.

**Methods:**

This study was a double-blinded randomized clinical trial, conducted on 67 patients with HNCs undergoing radiotherapy. The eligible participants were randomized to receive either one of the following; zinc sulfate, polyherbal, chlorhexidine (Vi-one 0.2% CHX), or placebo mouthwash for 6 weeks. Follow-up evaluation of oral hygiene and the checklists of OM and the intensity of pain were filled out according to WHO assessment tool, Oral Mucositis Assessment Scale (OMAS), and Visual Analog Scale (VAS) in all the participants weekly for seven consecutive weeks.

**Results:**

The results of present clinical trial demonstrated that the use of either zinc sulfate or polyherbal mouthwash significantly reduced the scores of OM and the severity of pain during weeks 2 to 7 after consumption compared with the CHX or placebo mouthwashes (*P* < 0.05). According to the post hoc analysis and compared with the placebo, a significantly better result was reported for zinc sulfate and polyherbal mouthwashes at weeks 2 to 7, but not for the CHX mouthwash.

**Conclusion:**

This study showed that the use of zinc sulfate or polyherbal mouthwashes is effective in prevention of both OM severity scores and pain related to OM intensity at weeks 2 to 7 following consumption in HNCs patients.

*Trial registration* IRCT20190123042475N1 and IRCT20190123042475N2. Registration date: 2019-06-09, 2019-07-26.

**Supplementary Information:**

The online version contains supplementary material available at 10.1186/s40001-023-01015-8.

## Introduction

Cancer is the second leading cause of mortality in the developed countries after cardiovascular diseases. Head and neck cancers (HNCs) are the 6th most common malignancies in the world [1, 2]. Head and neck cancers are related to malignant tumor cells in airways and upper digestive tract, including oral and nasal cavity, nasopharynx, oropharynx, hypopharynx, pharynx, larynx, middle ear and salivary glands [3, 4]. Based on the type and the stage of tumor, the patient may require one or a combination of the following modalities for the management of cancer: surgery, chemotherapy, and radiotherapy (RT) [5, 6]. Chemotherapy and RT are both the mainstay of treatment of head and neck cancers [7]. Adverse effect of RT depends on the radiation dose, type of ionizing irradiation, volume and extent of irradiated tissue, fraction size and the patient’s clinical condition [8, 9]. A significant percentage of HNCs patients receiving RT experience oral mucositis (OM), especially those with cumulative radiation dose > 30 Gy, [10, 11]. The prevalence of OM increases with simultaneous chemotherapy and radiotherapy [12]. Furthermore, some factors like age, sex, oral hygiene, smoking, alcohol consumption, human papillomavirus and diet predispose an individual to experience OM [4, 10].

OM is characterized by disruption of epithelial integrity in the oral cavity, pharynx and gastrointestinal tract [13]. The most common symptoms associated with OM are pain, dysphagia, dysgeusia, dehydration, anorexia, and weight loss. MO may interfere with cancer treatment and its efficacy. Thereby, this adverse effect of radiotherapy may increase length of hospitalization, adjuvant supportive care and treatment failure which all have a negative economic impact [14, 15]. Notably, the underlying pathophysiology of radiation-induced MO is presumed to be inflammation and reactive oxygen species (ROS) which are generated by ionizing radiation [7]. Various compounds have been proposed for prevention and management of radiation-induced OM [16]. However, until now, no proven prophylactic or treatment strategies have been globally accepted for OM [16].

Zinc has the potential to diminish oxidant injury caused by ROS [17]. It has a key role in collagen synthesis, fibroblast and keratinocyte proliferation [18]. Moreover, zinc is the cofactor of some cellular processes, such as synthesis of DNA, protein, RNA polymerase, and reverse transcriptase. Therefore, it has wound-healing properties while boosting the growth, immune and reproductive system [16, 19]. Chamomile (*Matricaria recutita L*.) is an herb used for its antibacterial, antifungal, inflammatory, angiogenesis, anti-carcinogenic, spasmolytic, antidiabetic and sedative properties for centuries [20, 21]. Peppermint oil is extracted from peppermint (*Mentha piperita L.*) plant. It has several active compounds such as terpenoids, flavonoids, polyphenol, alpha-tocopherol, betaine and choline [22]. Peppermint has multifunctional effects, better known for its analgesic and antimicrobial properties [23].

*Aloe vera* (*Aloe barbadensis Miller*) is an herbal medicine with valuable features. It is widely known for its anti-inflammatory, analgesic, immunoregulatory effects, and antimicrobial, wound healing, anti-proliferative and anti-tumor activities [24, 25]. There is a hypothesis stated that anti-inflammatory effect of *A. vera* is due to inhibition of cyclooxygenase, reduction of tumor necrosis factor alpha (TNF-α) levels and leukocyte adhesive molecule [26, 27]. Wound healing of *A. vera* arises from attenuation of vasoconstriction and platelet aggregation at wound site, increasing growth factors and collagen synthesis, enhancement of wound oxygenation, and neutralizing the free radicals [28, 29]. Honey has been used both as food and medicine from ancient times [30]. It has anti-inflammatory activity, antimicrobial, wound healing and reduction effect of free radicals [31, 32]. Moreover, honey contains an incredibly diverse range of nutrients to maintain a healthy weight in patient undergoing RT and/or chemotherapy [33, 34].

For prevention and management of OM, it is crucial to employ an efficient compound with likely safety profile which is easy to use. Currently, commercially therapeutic mouthwash on the market has high alcohol content, causing a burning sensation in the oral cavity [35]. They may also cause toxicity if swallowed or consumed in excess. In this clinical trial, we aimed to evaluate the effect of the polyherbal and zinc mouthwashes in comparison to control (chlorhexidine) and placebo groups for prevention of radiation-induced OM.

## Materials and methods

### Ethics considerations, setting, and patients

The study was approved by the Ethics and Research Committee of North Khorasan University of Medical Sciences Review Board. The trial was also registered and approved in the registry of clinical trials (IRCT20190123042475N1 and IRCT20190123042475N2). All the patients who took part in this study were provided informed consent prior to participation in the study. In this trial, we included patients over 18 years old undergoing RT with a minimum radiation dose of 30 Gy for the first time and when the oral cavity was in the range of radiation.

### Preparation and formulation of mouthwash

For the formulation of 1% zinc sulfate mouthwash, zinc sulfate was mixed with wetting agents (glycerin and water), stabilizers (ascorbic acid and citric acid) and microbial protection (potassium sorbate). For preparation of polyherbal mouthwash, chamomile extract, peppermint oil, *A. vera* gel, honey, potassium sorbate (as microbial protection), ascorbic acid and citric acid (stabilizers), glycerin and water (as wetting agents) were intermixed together. For formulation of placebo mouthwash, the same schedule was performed without adding the active ingredient composition. The placebo mouthwash contained all the ingredients used in the mouthwash of the intervention groups, except for the active ingredients, so to prepare the placebo mouthwash, citric acid, ascorbic acid, and potassium sorbate were mixed with glycerin and distilled water. The final formulations were then assessed for possible microbial contamination according to the United States Pharmacopeia at a research center of natural products and medicinal plants affiliated to the university.

### Sample size

The sample size was calculated considering a previous report by Mehdipour et al. [36]. We supposed to achieve a difference of 40% in obtaining the zero grade of OM in the intervention groups compared to the control and placebo groups after prescribing the intervention mouthwashes using the sample size equation (*N* = (z1 − α2/ − z1 − β) 2 (SD1 + SD2) 2/d2) for comparing the means. We also considered a confidence interval of 95%, statistically significant of 0.05, and statistical power of 80%. Therefore, the required sample size was calculated at least 15 patients per in each group, with a total sample size of 60 patients. The calculated sample size was increased to 67 to take into consideration the potential of loss to follow-up. A *p*-value of 0.05 or less was considered statistically significant.

### Randomization and blinding

Patients who met the inclusion criteria were randomized in a 1:1:1:1 ratio by a permuted block randomization method to one of the following groups: (1) zinc sulfate, (2) polyherbal, (3) CHX, or (4) placebo. So, blocks of four were applied and six-digit random numbers were generated. Patients were assigned to each of study group based on Random Number Table. To keep the concealment, the allocation list was given to a third person not involvedin the study.

To keep blinding, the mouthwashes were filled in identical bottles and coded by the principal investigator. Patients, radiation oncologist and investigator of clinical responses were all blind to the four arms of the study. When the weekly follow-up sessions were completed, the principal investigator decoded the random numbers of consumed bottles and assigned each to the proper arm.

### Patients and study procedure

This study was a double-blinded randomized clinical trial in 67 patients with HNCs undergoing RT in a tertiary referral hospital affiliated to medical university. After inspecting the oral cavity of the patients for any oral lesions, patients over 18 years of age with the oral mucosa within the range of radiation, and the minimum amount of radiation of 30 Gy were included in the study. At the initiation of the study, we assessed 75 patients for eligibility criteria. Eight patients were excluded from the study due to having one of the exclusion criteria: known allergy to the ingredients of the mouthwashes, previous history of receiving RT, use of any other mouthwash, consumption of oral zinc dietary supplements, or receiving analgesics. Eventually, statistical analysis was performed on the remaining 67 patients.

At baseline, just the day before the first RT session, the demographical and baseline characteristics of each patient were recorded by the investigator of clinical responses. The eligible participants were randomly assigned into one of the following groups: zinc sulfate, polyherbal, chlorhexidine (Vi-one 0.2% CHX), or placebo mouthwash. Prior to the enrollment, demographic characteristics of the patients were recorded. Afterwards, the oral cavity of all patients was examined. Follow-up evaluation of oral hygiene was performed in all the participants weekly for seven consecutive weeks. The checklists of OM and the intensity of pain were filled out according to WHO assessment tool [37], Oral Mucositis Assessment Scale (OMAS) [38], and Visual Analog Scale (VAS) [39]. Based on WHO classification system, OM is categorized into four stages. Score 0 indicates no signs or symptoms of OM. In score 1, the oral mucosa appears erythematosus and painful while in score 2, the oral cavity is ulcerous and the patient is unable to eat normally. Score 3 is described when the ulcer has progressed to some extent that the patient can only drink fluid. In score 4, the patient cannot even drink fluid [40]. The OMAS criterion evaluates ulceration and erythema at nine sites of the oral cavity. In each site of this cavity, the presence of an ulceration is scored from 0 to 3 as follows: score 0 (no ulceration), score 1 (the cumulative surface area of the ulcer < 1 cm2), score 2 (1 cm2 ≤ the surface area ≤ 3 cm2). score 3 (the surface area > 3 cm2). Erythema is also scored from 0 to 2: score 0 (no redness), score 1 (mild to moderate redness), and score 2 (severe erythema with the color of fresh blood). Eventually, these scores in all nine parts of the oral cavity are then summed up and the average score is reported between 0 (absence of mucositis) and 5 (the worst severity of mucositis) [38]. The Visual Analog Scale is a means to measure the intensity of pain that is scored by the patient as a self-report. In this criterion, the intensity of pain is scored from 0 to 10, so that score 0 indicates the absence of pain and a score of 10 indicates the intensity of pain that is intolerable [41].

Each patient was instructed to rinse 5 ml of the mouthwash three times a day for 60 s from the first day of starting RT, then poured it out. The patients were informed to continue their treatment for the following 6 weeks. Patients were evaluated weekly during RT by radiation oncologist and investigator of clinical responses. In these weekly follow-up sessions, mouthwash was provided to the patients for one week. Compliance to the treatment was assessed by requesting the patients to take the rest of their bottles to the weekly appointments. The oral cavity was examined and the incidence and severity of OM and intensity of pain was evaluated on a weekly basis (for an overall of seven follow-up sessions).

### Statistical analyses

Data analysis was performed using SPSS 23 software. We have used Chi-square test or Fisher exact test for comparing two qualitative variables. For comparing groups according to quantitative variables (as our quantitative variables did not have normal distribution), we have used Kruskal–Wallis test. Post hoc analysis was used to assess the difference between any pair of group means. In all cases, *P-value* < 0.05 was considered as statistically significant difference.

## Results

The current study is a double-blind, randomized clinical trial in 67 patients with HNCs. Among 75 patients assessed for eligibility, 67 were enrolled to receive either of interventions: zinc sulfate, polyherbal (chamomile, peppermint oil, *A. vera*, and Honey), CHX or placebo mouthwash. The baseline demographic and clinical characteristics of patients are presented in Table 1 and appeared to be comparable.

**Table 1:** Characteristics of the patients at baseline (*n* = 67)

Characteristic	Groups				*P-value*
	CHX	Polyherbal	Zinc sulfate	Placebo	
Patients enrolled (*n*)	17	17	17	16	
Men, *N* (%)	10 (58.8)	10 (58.8)	8 (47.1)	10 (62.5)	0.824
Age (y), mean (SD)	68.7 (8.5)	67.4 (9.2)	65.8 (11.2)	66.9 (10.1)	0.633
BMI (kg/cm^2^), mean (SD)	23.0 (3.8)	22.6 (4.8)	22.1 (5.2)	23.1 (5.5)	0.972
Occupation, *N* (%)					
Farmer	3 (17.6)	3 (17.6)	2 (11.8)	2 (12.5)	0.865
Laborer	0 (0.0)	1 (5.9)	2 (11.8)	2 (12.5)	
Retired	1 (5.9)	2 (11.8)	0 (0.0)	3 (18.8)	
Self-employment	4 (23.5)	3 (17.6)	4 (23.5)	3 (18.8)	
Housekeeper	7 (41.2)	7 (41.2)	9 (52.9)	6 (37.5)	
Other	2 (11.8)	1 (5.9)	0 (0.0)	0 (0.0)	
Educational status, *N* (%)					
Illiterate	9 (52.9)	5 (29.4)	11 (64.7)	6 (37.5)	0.291
Elementary	7 (41.2)	8 (47.1)	4 (23.5)	7 (43.8)	
Diploma	0 (0.0)	3 (17.6)	2 (11.8)	3 (18.8)	
BS/AS	1 (5.9)	1 (5.9)	0 (0.0)	0 (0.0)	
Type of tumor, *N* (%)					
Larynx	4 (23.5)	3 (17.6)	4 (23.5)	5 (31.3)	0.983
BCC	4 (23.5)	5 (29.4)	4 (23.5)	5 (31.3)	
Neck	1 (5.9)	2 (11.8)	3 (17.6)	1 (6.3)	
Saliva glands	5 (29.4)	3 (17.6)	2 (11.8)	2 (12.5)	
SCC	3 (17.6)	4 (23.5)	4 (23.5)	3 (18.8)	
Smoking, *N* (%)					
Yes	8 (47.1)	4 (23.5)	6 (35.3)	7 (43.8)	0.529
No	9 (52.9)	13 (76.5)	11 (64.7)	9 (56.3)	
Dentures, *N* (%)					
Yes	14 (82.3)	12 (70.6)	10 (58.8)	12 (75.0)	0.089
No	2 (17.7)	5 (29.4)	7 (41.2)	4 (25.0)	
History of surgery, *N* (%)					
Yes	5 (29.4)	7 (41.2)	7 (41.2)	4 (25.0)	0.721
No	12 (70.6)	10 (58.8)	10 (58.8)	12 (75.0)	
Cumulative radiation dose (Gy), mean (SD)	6110.5 (623.6)	6169.4 (729.6)	6404.7 (623.6)	6238.7 (687.9)	0.677
Radiotherapy cycle, *N* (%)					
25	1 (5.9)	3 (17.6)	3 (17.6)	2 (12.5)	0.628
30	6 (35.4)	6 (35.4)	7 (41.3)	5 (31.2)	
33	2 (11.8)	2 (11.8)	2 (11.8)	3 (18.8)	
35	8 (47.1)	6 (35.4)	5 (29.5)	6 (37.5)	

*N* number; *y* year; *BMI* body mass index; *CHX* chlorhexidine; *BCC* basal cell carcinoma; *SCC* squamous cell carcinoma; *cm* centimeter; *kg* kilogram; *SD* standard deviation; *Gy* gray

### Outcomes

The details of the severity of OM are presented in Table 2 according to WHO scale over week 1 to 7 for CHX, polyherbal, zinc sulfate, and placebo groups. At study termination (week 7), most patients in the zinc sulfate group were free of symptoms (8 out of 17 patients, 47.1%). Then after, herbal group took the second place with 41.1% of patients free of symptoms (*P*-value = 0.001). Patients in the placebo group had the highest grade 3 experience of OM (56.25%). None of the patients in each of four groups experienced grade 4 of OM during the 7 weeks of the study period. The severity of mucositis increased significantly in placebo group as RT continued, compared with each of intervention groups (Additional file 1: Table S1).

**Table 2:** The severity of oral mucositis according to WHO scale over time during weeks 1 to 7 for CHX, polyherbal, zinc sulfate, and placebo groups

Week	Grade	Groups					*P-value*
		CHX	Herbal	Zinc sulfate	Placebo	Total	
1	0	17 (25.4)	17 (25.4)	17 (25.4)	16 (23.8)	67 (100.0)	1.000
	1	0 (0.0)	0 (0.0)	0 (0.0)	0 (0.0)	0 (0.0)	
	2	0 (0.0)	0 (0.0)	0 (0.0)	0 (0.0)	0 (0.0)	
	3	0 (0.0)	0 (0.0)	0 (0.0)	0 (0.0)	0 (0.0)	
	4	0 (0.0)	0 (0.0)	0 (0.0)	0 (0.0)	0 (0.0)	
2	0	13 (19.4)	17 (25.4)	16 (23.9)	8 (11.9)	54 (80.6)	**0.001**^**a**^
	1	3 (4.8)	0 (0.0)	1 (1.5)	5 (7.5)	9 (14.43)	
	2	1 (1.6)	0 (0.0)	0 (0.0)	3 (4.5)	4 (6.0)	
	3	0 (0.0)	0 (0.0)	0 (0.0)	0 (0.0)	0 (0.0)	
	4	0 (0.0)	0 (0.0)	0 (0.0)	0 (0.0)	0 (0.0)	
3	0	9 (14.43)	13 (19.4)	12 (17.9)	5 (7.5)	39 (58.2)	**0.006**^**a**^
	1	3 (4.8)	4 (6.0)	4 (6.0)	3 (4.5)	14 (20.9)	
	2	5 (7.6)	0 (0.0)	1 (1.6)	7 (10.5)	13 (19.4)	
	3	0 (0.0)	0 (0.0)	0 (0.0)	1 (1.5)	1 (1.5)	
	4	0 (0.0)	0 (0.0)	0 (0.0)	0 (0.0)	0 (0.0)	
4	0	5 (7.6)	10 (15.1)	10 (15.15)	2 (3.0)	27 (40.9)	** < 0.001**^**a**^
	1	5 (7.7)	6 (9.1)	5 (7.6)	2 (3.0)	18 (27.3)	
	2	6 (9.1)	1 (1.6)	2 (3.2)	8 (12.1)	17 (25.8)	
	3	1 (1.6)	0 (0.0)	0 (0.0)	3 (4.5)	4 (6.0)	
	4	0 (0.0)	0 (0.0)	0 (0.0)	0 (0.0)	0 (0.0)	
5	0	2 (3.2)	7 (10.8)	8 (12.3)	1 (1.5)	18 (27.7)	**0.001**^**a**^
	1	4 (6.0)	4 (6.0)	4 (6.0)	2 (3.0)	14 (21.5)	
	2	5 (7.7)	6 (9.2)	4 (6.0)	6 (9.2)	21 (32.3)	
	3	5 (7.7)	0 (0.0)	1 (1.6)	6 (9.2)	12 (18.5)	
	4	0 (0.0)	0 (0.0)	0 (0.0)	0 (0.0)	0 (0.0)	
6	0	1 (1.6)	7 (11.3)	8 (12.9)	1 (1.5)	17 (27.4)	**0.001**^**a**^
	1	3 (4.8)	4 (6.4)	3 (4.8)	1 (1.5)	11 (17.7)	
	2	3 (4.8)	4 (6.4)	4 (6.4)	4 (6.4)	15 (24.2)	
	3	6 (9.7)	2 (3.2)	2 (3.2)	9 (14.5)	19 (30.6)	
	4	0 (0.0)	0 (0.0)	0 (0.0)	0 (0.0)	0 (0.0)	
7	0	1 (1.6)	7 (11.3)	8 (12.9)	1 (1.5)	17 (27.4)	**0.001**^**a**^
	1	3 (4.8)	4 (6.0)	3 (4.8)	1 (1.5)	11 (17.7)	
	2	3 (4.8)	4 (6.0)	4 (6.4)	4 (6.4)	15 (24.2)	
	3	6 (9.7)	2 (3.2)	2 (3.2)	9 (14.5)	19 (30.6)	
	4	0 (0.0)	0 (0.0)	0 (0.0)	0 (0.0)	0 (0.0)	

Data are based on number (%)

The bold values in this table indicates statistically significant difference between four study groups in weeks 2–7, according to WHO scale

^a^Statistically significant

The severity of oral mucositis according to OMAS over time is presented in Table 3. The results obtained from OMAS in the severity of OM were in line with the results of WHO scale. Any presented lesions were measured and scored in OMAS. As illustrated, both the size and redness of lesions were reduced significantly in each of interventional groups at weeks 2 to 7 compared to the placebo group.

**Table 3:** Severity of oral mucositis according to OMAS over time during weeks 1 to 7 for CHX, polyherbal, zinc sulfate, and placebo groups

Week	Groups				*P*-value
	CHX	Herbal	Zinc sulphate	Placebo	
1	0.00 ± 0.00	0.00 ± 0.00	0.00 ± 0.00	0.00 ± 0.00	1.000
2	0.35 ± 0.78	0.00 ± 0.00	0.06 ± 0.24	0.94 ± 1.29	**0.001**^**a**^
3	1.06 ± 1.34	0.23 ± 0.44	0.41 ± 0.79	2.62 ± 2.61	**0.003**^**a**^
4	2.06 ± 2.01	0.65 ± 0.99	0.82 ± 1.28	4.06 ± 3.11	** < 0.001**^**a**^
5	3.31 ± 2.67	1.58 ± 1.66	1.47 ± 2.03	5.00 ± 3.36	**0.001**^**a**^
6	4.37 ± 2.92	2.12 ± 2.06	2.23 ± 2.82	6.20 ± 3.23	**0.001**^**a**^
7	5.06 ± 2.84	2.58 ± 2.576	2.71 ± 3.33	6.86 ± 3.74	**0.002**^**a**^

Data are based on mean ± SD

The bold values in this table indicates statistically significant difference between four study groups in weeks 2-7, according to OMAS scoring system

^a^Statistically significant

The mean severity of pain in study groups were evaluated by using VAS score (Table 4). The mean (SD) severity of pain in polyherbal group in 2th, 3th, 4th, and 5th weeks were lower than the others groups while the mean severity of pain in zinc sulfate group were lower in 6th and 7th weeks.

**Table 4:** The mean severity of oral mucositis related pain according to VAS score over time during weeks 1 to 7 for CHX, polyherbal, zinc sulfate, and placebo groups

Week	Groups				*P-value*
	CHX	Herbal	Zinc sulfate	Placebo	
1	0.00 ± 0.00	0.00 ± 0.00	0.00 ± 0.00	0.00 ± 0.00	1.000
2	0.76 ± 1.36	0.00 ± 0.00	0.29 ± 1.21	1.62 ± 1.75	**0.002**^**a**^
3	1.58 ± 2.06	0.71 ± 1.36	1.00 ± 1.69	3.00 ± 2.28	**0.009**^**a**^
4	3.00 ± 2.29	1.53 ± 1.97	1.71 ± 2.26	4.33 ± 2.28	**0.005**^**a**^
5	4.68 ± 2.82	2.42 ± 2.45	2.47 ± 2.67	5.13 ± 2.19	**0.006**^**a**^
6	6.06 ± 2.46	3.12 ± 2.85	2.94 ± 3.07	6.40 ± 2.47	**0.001**^**a**^
7	6.94 ± 2.62	4.00 ± 3.61	3.58 ± 3.71	7.60 ± 2.58	**0.001**^**a**^

Data are based on mean ± SD

The bold values in this table indicates statistically significant difference between four study groups in weeks 2-7, according to VAS scale

^a^Statistically significant

Post hoc analysis, comparing the reduction in WHO, OMAS, and VAS scores at weeks 2 to 7 from baseline indicated no statistically significant in the zinc sulfate group vs polyherbal group and the CHX group vs placebo group (*P-value* > 0.05). Additional post hoc analysis showed a significant reduction in each of WHO, OMAS, and VAS scores in either zinc sulfate or polyherbal groups compared with the placebo group (*P-value* < 0.05). The results of the post hoc analysis of comparing zinc sulfate and polyherbal groups with the CHX group were varied. The reduction in VAS score were significant at weeks 5 to 7 for each of zinc sulfate or polyherbal groups vs CHX group, while the changes in either WHO or OMAS scores were significant at weeks 4 to 7 (Additional file 1: Table S1).

## Discussion

The current double-blind, randomized clinical trial aimed to evaluate the effect of zinc sulfate and polyherbal (chamomile, peppermint oil, *A. vera*, and honey) mouthwashes compared with each of two control groups (CHX and placebo) in prevention of radiation-induced oral mucositis in patients with HNC. The results of this trial showed that the use of either zinc sulfate or polyherbal mouthwash significantly reduced the scores of OM and the severity of pain during weeks 2–7 after consumption compared with the CHX or placebo mouthwashes. According to the post hoc analysis and compared with the placebo, a significantly better result was reported for zinc sulfate and polyherbal mouthwashes at weeks 2–7, but not for the CHX mouthwash.

The polyherbal mouthwash formulated in this study contained chamomile extract, *A. vera* gel, peppermint oil, and honey, and the effectiveness of each component in this mouthwash has been evaluated in different clinical studies in OM. Honey, a natural product with wound-healing properties, prevents microbial growth due to high viscosity and osmolality along with the low PH. Natural honey stimulates the saliva secretion, and activates immune system responses [33]. Moreover, it has anti-inflammatory and antioxidant effects by decreasing the levels of prostaglandin and increasing nitric oxide levels in the lesional sites, so it accelerates the healing of acute and chronic wounds [42]. Favorable taste and lack of cytotoxic effect on host cells increase the compliance of using honey as a mouthwash in patients experiencing RT-induced OM [31]. The efficacy of topical honey has been shown in randomized clinical trials in preventing the development and severity of OM, the growth of aerobic bacteria and candida, attenuation the intensity of pain, and improving the quality of life [31, 43–45].

Another key component in polyherbal mouthwash was chamomile extract. The importance of flavonoids in the chamomile plant and the antibacterial, antifungal, and anti-inflammatory effects of this plant along with its low price and accessibility made it a distinctive herb in the pharmaceutical industry [46]. The use of chamomile liquid oral rinse in patients undergoing radiochemotherapy has the ability to delay the onset and severity of RT-induced mucositis compared with placebo [47].

Aloe vera was also applied in the polyherbal mouthwash formulated in the present study. *A. vera* is a medicinal plant, containing over 75 active constituents, which makes it a suitable choice for wound healing [24]. It appears that *A. vera* exerts its favorable effects in wound healing process through inhibition of cyclooxygenase, improvement of collagen synthesis and blood flow to the wound site, scavenging the free radicals, and antibacterial properties [28]. *A. vera* has shown to significantly reduce the incidence of sever RT-induced mucositis in patients with HNCs [48].

Peppermint has long been used as a cooling agent in topical pharmaceutical products. Peppermint oil has antioxidant and antimicrobial (bacteria, viruses and parasites) properties [49]. This compound has also been used in the prevention of mucositis in previous studies. The combination of *Matricaria recutita* and *Mentha piperita* in patients with hematopoietic stem cell transplantation significantly diminished the duration, the maximum and the average daily grade of OM compared with placebo. Moreover, the intensity of pain, dryness, and dysphagia were significantly lower in intervention group [50]. Therefore, it appears that a polyherbal product containing all of these components can potentially prevent this complication of radiotherapy. The results of this study showed that the severity of oral mucositis based on both WHO and OMAS scales, as well as pain based on VAS, was significantly lower in polyherbal group compared to the placebo group. In the present study, the patients in the polyherbal group did not experienced even grade 3 OM throughout the study. The overall OMAS score was lower in polyherbal group in comparison to the other groups.

The role of zinc sulfate has been shown previously in acceleration of wound healing process [17, 18]. This compound has been used both as an oral supplement and as a mouthwash for prevention of OM. The use of this agent has reduced the onset of this complication and the severity oral mucositis due to RT in HNC patients. Zinc sulfate could diminish the onset and severity of OM in HNC patients undergoing radiation, especially at weeks 4 to 6 [16, 51]. However, the results regarding the effectiveness of zinc sulfate are contradictory, since this component has not always been able to reduce the severity of mucositis and esophagitis in RT related OM [52]. In the current clinical trial, the recipient of zinc sulfate mouthwash showed a significant difference in the occurrence and severity of OM compared with both placebo and CHX groups.

Although the findings of the present RCT suggest that either zinc sulfate or polyherbal (containing chamomile, peppermint oil, *A. vera*, and honey) mouthwashes are potential preventive candidates for RT-induced OM in patients with HNCs, the limitations of the study should be considered and care should be taken in interpreting the results. The first limitation, due to the small sample size, is the generalizability of the results of the present study to all patients undergoing radiotherapy, which should be confirmed in a larger group of HNCs patients in future studies. Second, given that the effects of polyherbal mouthwash were evaluated in combination, it is not clear which component of the mouthwash was responsible for the beneficial reported effects. Furthermore, since the product was formulated as mouthwash, its efficacy was limited to only the oral cavity, while the radiotherapy-induced mucositis can also involve the mucous membranes in deeper areas. Although head and neck cancer was the main inclusion criteria for patients to enroll into the study, the type of cancer was unfortunately not recorded in the data collection form. Therefore, we were unable to perform subgroup analysis in this regard to figure out which type of HNCs caused the most scoring of oral mucositis.

## Conclusion

The use of zinc sulfate or polyherbal (containing chamomile, peppermint oil, *A. vera*, and honey) mouthwashes in patients with HNCs showed a significant reduction in OM severity scores and the related pain at weeks 2–7. However, further evaluation with larger sample size is warranted to assess their safety and efficacy in this population of patients.

## Supplementary Information

**Additional file 1: Table S1.** Post hoc analysis on pairwise comparisons between the groups.

## Data Availability

The datasets used and/or analyzed during the current study available from the corresponding author on reasonable request. The raw SPSS file of this study before analysis is available upon your request.

## Abbreviations

HNCs: Head and neck cancer
OM: Oral mucositis
CHX: Chlorhexidine
OMAS: Oral Mucositis Assessment Scale
VAS: Visual analog scale
RT: Radiotherapy
ROS: Reactive oxygen species
A. vera: Aloe vera
TNF-α: Tumor necrosis factor alpha
N: Number
y: Year
BMI: Body mass index
BCC: Basal cell carcinoma
SCC: Squamous cell carcinoma
SD: Standard deviation
Gy: Gray
