# Supplementary material for: Prevention of radiotherapy-related oral mucositis with zinc and polyherbal mouthwash: a double-blind, randomized clinical trial
Source: Eur J Med Res. 2023 Mar 2;28:109. doi: 10.1186/s40001-023-01015-8 (PMC9979417; doi:10.1186/s40001-023-01015-8)
Supplement: Supplementary file 1 — Additional file 1: Table S1. Post hoc analysis on pairwise comparisons between the groups. [file 40001_2023_1015_MOESM1_ESM.docx]

| Table S1. Post-hoc analysis on pairwise comparisons between the groups. | | | | | | | | | |
| --- | --- | --- | --- | --- | --- | --- | --- | --- | --- |
| **Comparison** | | Severity of oral mucositis according to WHO scale | | | | | | | |
|  |  | Week | 1 | 2 | 3 | 4 | 5 | 6 | 7 |
| Placebo | CHX | Comparison, mean | 0.000 | 2.578 | 1.986 | 3.374 | 0.763 | 1.438 | 1.438 |
|  |  | P-value | 1.000 | 0.108 | 0.159 | 0.066 | 0.382 | 0.230 | 0.230 |
|  | Herbal | Comparison, mean | 0.000 | 10.686 | 9.512 | 13.128 | 9.927 | 10.112 | 10.112 |
|  |  | P-value | 1.000 | 0.001 | 0.002 | 0.000 | 0.002 | 0.001 | 0.001 |
|  | Zinc sulfate | Comparison, mean | 0.000 | 8.057 | 7.246 | 11.772 | 9.680 | 10.417 | 10.417 |
|  |  | P-value | 1.000 | 0.005 | 0.007 | 0.001 | 0.002 | 0.001 | 0.001 |
| CHX | Herbal | Comparison, mean | 0.000 | 4.387 | 3.200 | 5.144 | 5.388 | 6.512 | 6.512 |
|  |  | P-value | 1.000 | 0.036 | 0.074 | 0.023 | 0.020 | 0.011 | 0.011 |
|  | Zinc sulfate | Comparison, mean | 0.000 | 2.121 | 1.827 | 4.245 | 5.789 | 6.916 | 6.916 |
|  |  | P-value | 1.000 | 0.145 | 0.177 | 0.039 | 0.016 | 0.009 | 0.009 |
| Herbal | Zinc sulfate | Comparison, mean | 0.000 | 1.000 | 0.222 | 0.019 | 0.076 | 0.040 | 0.040 |
|  |  | P-value | 1.000 | 0.317 | 0.638 | 0.890 | 0.783 | 0.841 | 0.841 |
|  |  | Severity of oral mucositis related pain according to VAS score | | | | | | | |
| Placebo | CHX | Comparison, mean | 0.000 | 2.685 | 3.296 | 2.667 | 0.103 | 0.159 | 0.853 |
|  |  | P-value | 1.000 | 0.101 | 0.069 | 0.102 | 0.748 | 0.690 | 0.356 |
|  | Herbal | Comparison, mean | 0.000 | 10.654 | 9.125 | 9.898 | 7.651 | 9.075 | 8.943 |
|  |  | P-value | 1.000 | 0.001 | 0.003 | 0.002 | 0.006 | 0.003 | 0.003 |
|  | Zinc sulfate | Comparison, mean | 0.000 | 6.635 | 6.709 | 7.462 | 7.149 | 8.638 | 9.311 |
|  |  | P-value | 1.000 | 0.010 | 0.010 | 0.006 | 0.008 | 0.003 | 0.002 |
| CHX | Herbal | Comparison, mean | 0.000 | 4.381 | 1.719 | 3.526 | 5.131 | 7.699 | 5.213 |
|  |  | P-value | 1.000 | 0.036 | 0.190 | 0.060 | 0.024 | 0.006 | 0.022 |
|  | Zinc sulfate | Comparison, mean | 0.000 | 1.726 | 0.724 | 2.369 | 4.835 | 7.516 | 6.270 |
|  |  | P-value | 1.000 | 0.189 | 0.395 | 0.124 | 0.028 | 0.006 | 0.012 |
| Herbal | Zinc sulfate | Comparison, mean | 0.000 | 1.000 | 0.218 | 0.045 | 0.001 | 0.027 | 0.131 |
|  |  | P-value | 1.000 | 0.317 | 0.641 | 0.831 | 0.971 | 0.870 | 0.718 |
|  |  | Severity of oral mucositis according to OMAS scale | | | | | | | |
| Placebo | CHX | Comparison, mean | 0.000 | 2.642 | 3.675 | 3.837 | 2.008 | 2.542 | 2.123 |
|  |  | P-value | 1.000 | 0.104 | 0.055 | 0.050 | 0.156 | 0.111 | 0.145 |
|  | Herbal | Comparison, mean | 0.000 | 10.676 | 9.876 | 12.798 | 10.374 | 11.120 | 9.184 |
|  |  | P-value | 1.000 | 0.001 | 0.002 | 0.000 | 0.001 | 0.001 | 0.002 |
|  | Zinc sulfate | Comparison, mean | 0.000 | 8.050 | 8.094 | 11.272 | 10.530 | 9.499 | 8.368 |
|  |  | P-value | 1.000 | 0.005 | 0.004 | 0.001 | 0.001 | 0.002 | 0.004 |
| CHX | Herbal | Comparison, mean | 0.000 | 4.387 | 3.200 | 5.206 | 3.890 | 4.853 | 5.230 |
|  |  | P-value | 1.000 | 0.036 | 0.074 | 0.023 | 0.049 | 0.028 | 0.022 |
|  | Zinc sulfate | Comparison, mean | 0.000 | 2.121 | 1.827 | 4.011 | 4.991 | 4.963 | 4.821 |
|  |  | P-value | 1.000 | 0.145 | 0.177 | 0.045 | 0.025 | 0.026 | 0.028 |
| Herbal | Zinc sulfate | Comparison, mean | 0.000 | 1.000 | 0.222 | 0.031 | 0.146 | 0.012 | 0.008 |
|  |  | P-value | 1.000 | 0.317 | 0.638 | 0.860 | 0.703 | 0.914 | 0.928 |

Chi-Square test was used to compare these values.
